# Generative AI and medical oncologists: Be curious, but cautious!

**DOI:** 10.1177/03008916251388549

**Published:** 2026-01-08

**Authors:** Silvana Quaglini, Lucia Sacchi, Federico Sottotetti, Andrea Premoli, Laura D. Locati

**Affiliations:** 1 Department of Electrical, Computer and Biomedical Engineering, University of Pavia, Italy; 2 Medical Oncology Unit, Istituti Clinici Scientifici Maugeri IRCCS, Pavia, Italy; 3 Department of Internal Medicine and Therapeutics, University of Pavia, Italy

**Keywords:** ChatGPT, artificial intelligence (AI), oncology, large language models (LLMs), medical decision-making, AI hallucinations

## Abstract

The purpose of this editorial is to explore the potential and risks of using AI in the medical field, particularly in oncology. We describe the history of AI from its origins to the present day, highlighting its positive aspects and potential for each type of AI (expert system, machine learning, generative AI). Technologies like ChatGPT are increasingly being used across various domains; however, without proper caution, they can give rise to phenomena such as 'AI hallucinations'—responses that may appear precise, detailed, clear, and logical, but are in fact entirely fabricated and unfounded, potentially confusing those who read them. We want to caution oncologists against the unconditional use of these technologies, where human input remains still essential both in interpreting the responses and in formulating the questions.

## Generative AI and medical oncologists

Generative Artificial Intelligence (AI) tools, particularly those based on Large Language Models (LLMs), have become increasingly popular among physicians, including oncologists, making it essential to highlight how they are changing medical practice. In recent discussions about the use of Generative AI tools to support diagnostic or therapeutic decision-making, we have observed that physicians tend to be more enthusiastic than biomedical engineers or computer scientists. This is likely because a deeper understanding of how these tools operate allows the latter to better anticipate the potential risks of indiscriminate use. For this reason, we claim that educating and training physicians in the fundamental principles of these technologies, even at a high level, is crucial to prevent misuse. Before focusing on generative AI, since a lot of systems nowadays are labelled as “AI systems”, it is important to point out that AI is a very broad umbrella encompassing various paradigms, as also defined in the *AI Act* by the European Union.^1^ In its history, AI has gone through both periods of great enthusiasm and dark periods, so-called “AI winters”, times of reduced interest and funding.^2^ The definition of AI was first formulated by John McCarthy in 1955 in terms of "the science and engineering of making intelligent machines, especially intelligent computer programs". It's also worth noting that many of the techniques used in AI have existed since the 1950s or even earlier. In the following sections, we highlight key milestones in the adoption of AI techniques in medicine. Below, we describe the three main paradigms of AI from the 1970s to the present. Their definitions, along with their main advantages and disadvantages, are summarized in Table 1.

**Table 1.** Definition, Pros and Cons of the different AI paradigms.

	Different types of AI		
	Expert Systems	Machine Learning and Deep Learning	Generative AI
Why it is defined as AI	System emulates human reasoning, by matching a patient’s data with an explicitly represented knowledge base	System automatically learns a predictive model from data	System can chat about everything in real time and generates its answers automatically in a format that is indistinguishable from human responses
Pros	Its performance can be tested, as the results are fully verifiable, repeatable, and explainable. Medical Device regulation is applicable	The model is developed automatically, reducing time and manual effort, i.e., no need to formalize explicit rules. Some models, e.g., logistic regression, are explainable. Medical Device regulation is sometime applicable	The model can generate responses on a variety of topics, though this does not guarantee complete reliability or accuracy. The interaction is very user-friendly and self-adapting to the user literacy
Cons	The *closed-world assumption*, meaning decision support is limited to the domain represented by the rules. Significant amount of time required to write the rules in a computational language. Maintenance is demanding, as the system must be updated whenever new scientific evidence becomes available	Generalizability is not guaranteed. Performance heavily depends on the quality of the training datasets. If the data quality is poor, the model may learn incorrect patterns. Most machine learning (ML) and deep learning (DL) models are “black boxes,” meaning the results are not easily explainable.	Over-reliance on the system’s answers: there is a risk of placing too much confidence in the system’s answers, as the response could be very well phrased, but entirely wrong or only partially correct. No regulation available yet for their medical use.

The first significant era of AI in medicine began in the early 1970s with the development of so-called *expert systems*, notably the seminal work by Shortliffe et al.^3^ An *expert system* is a computerized collection of several explicit rules (e.g., “IF Hemoglobin < 13g/dL AND sex = Female THEN diagnosis = Anemia”) that represent medical knowledge. When matched with a patient’s data, these rules can be used to emulate the reasoning processes of medical experts. Within an expert system, the reasoning process is performed by an *inference engine*, which may operate using different approaches. For example, the *inference engine* described by Lanzola et al.^4^ implements an epistemological model for anemia diagnosis. This model supports multiple forms of inference: *abstraction*, to derive higher-level concepts from raw data (e.g., identifying temporal trends in lab values); *abduction*, for generating diagnostic hypotheses; *deduction*, for testing those hypotheses; and *eliminative induction*, for refining them.^5^ The results produced by these systems are deterministic, although the combination of rules may involve complex calculations, including uncertainty coefficients associated with the conclusions. This rule-based paradigm remains in use today in decision support systems based on computerized clinical practice guidelines. These systems may be designed for physicians^6,7^ or for patients,^8,9^ with the latter often delivered through telemedicine platforms. The validation process^10^ developed and refined over the years has also facilitated the certification of such systems as medical devices. Examples are the Deontics Composer (http://deontics.com/technology) and Cureety TechCare, a telemonitoring medical device for oncology (http://www.cureety.com/en/patient-en/).

In the late 1990s, the increasing availability of electronic medical data^11^ accelerated the rise of the second paradigm of AI: Machine Learning (ML). Over the years, this has been increasingly strengthened by the introduction of innovative tools for data storage and integration, such as i2b2 (Informatics for Integrating Biology and the Bedside)^12^ and REDcap (Research Electronic Data Capture),^13^ as well as increased computational power. ML models primarily perform classification tasks, i.e., they can take a patient’s data as input and assist clinicians in selecting a diagnosis or treatment from a predefined set of options. This data-driven AI paradigm relies on models that are able to learn their parameters, in some cases even their structure, from a set of given “examples”. A model is trained on a dataset (a *training set* of already labelled or *classified* examples) using *supervised classification*, where both the input data and the corresponding correct classification are available. For example, a set of biomedical images, each one labelled as “positive” or “negative” according to the presence or absence of a tumor mass as annotated by a clinical expert, can be used as a training set for a ML model. The model’s parameters are usually optimized (or fine-tuned) using a *validation set*, which may also be used to choose among a set of candidate models the one that performs best. Its performance and generalization power are subsequently evaluated on a third, independent *test set*. Well-known ML models^14^ are decision trees, random forests, support vector machines and neural networks. However, also more “classical” models like Bayesian (causal) networks or even logistic regression can be used for ML, and they are favored by a school of thought that privileges explainable models, claiming that strong AI requires knowledge about causal processes.^15^

Starting in the early 2000s, due to technological advancements, ML began to incorporate more complex models, eventually leading to the rise of Deep Learning (DL). This third paradigm represents a significant breakthrough. While traditional ML typically requires preliminary, often human-driven, processing of data for feature extraction, DL models can automatically extract relevant features from raw data during the training process. DL is based on multi-layer neural networks and requires very large amounts of training data and substantial computational power to perform effectively. Highly successful DL-based systems have been developed, particularly in the fields of omics^16^ and biomedical image interpretation.^17^ However, the black-box nature and low interpretability of most ML and DL systems make their certification as medical devices a challenging task, even though several certified tools already exist.^18^ Ongoing efforts in explainability (also referred to as trustworthy AI)^19,20^ are expected to help overcome these challenges and support the certification process. Beginning in the late 2010s, a specific deep learning architecture known as the Transformer, sparked the revolution we are currently witnessing in the field of AI.^21^ This development marks a significant paradigm shift toward what is now referred to as Generative AI. Large Language Models (LLMs), the foundation of widely known tools such as ChatGPT (OpenAI), Gemini (Google), Claude (Anthropic), and others,^22^ are essentially deep neural networks that take a sequence of words (the *prompt*, i.e., the user’s input) and produce another sequence of words as output. LLMs sharing the principle of neural networks as a reasoning model, are not free from the same issues as DL and ML when it comes to certifying the system, as these models are difficult to interpret. Oversimplifying somewhat, since the underlying algorithms are highly complex, LLMs generate responses by selecting the next word in a sequence to maximize the probability of that word occurring in that specific context. In essence, the mechanism is purely statistical. These systems do not rely on explicitly encoded knowledge or structured datasets, nor are they trained on labeled relational datasets, which are typical in supervised learning. Rather, again simplifying, they are trained on massive collections of text documents and web content, that can range from completely unstructured to carefully organized and curated, such as document collections or indexed web pages which are used to build their deep architectures, characterized by an extremely large number (currently billions) of parameters. Generative AI thus operates by capturing statistical correlations between formal symbols, but without knowing their semantics, generating an appearance of meaning similar to what Searle described in the "Chinese Room" argument.^23^ Because of this underlying mechanism, the generated answers are always plausible but not necessarily correct. In fact, AI can produce responses that appear precise, detailed, clear, and logical, but these can be completely fabricated and unfounded, a phenomenon known as *AI hallucinations*.

We observed an example of this phenomenon during an analysis conducted to evaluate ChatGPT-4 performance in selecting the most appropriate treatment for breast cancer patients, based on the guidelines of the Italian Association of Medical Oncology (AIOM) for early-stage breast cancer. A series of clinical questions were prepared and submitted to ChatGPT to obtain treatment suggestions. Four oncologists (three experts and one resident) evaluated the AI-generated responses using the following criteria: (1) compliance with the guidelines; (2) overall quality of the response; (3) appropriateness of the answer; (4) verbosity; and (5) clinical actionability. Each criterion was rated using a Likert scale.^24^ In this evaluation, ChatGPT-4 occasionally incorrectly recommended aromatase inhibitors for a subset of patients with ductal carcinoma in situ (DCIS), even though these patients are not eligible for that treatment. This likely occurred because aromatase inhibitors are frequently cited in the guidelines as the recommended treatment for estrogen receptor-positive breast cancer. However, the model failed to recognize that for patients with DCIS, this treatment is explicitly not recommended, as stated in the AIOM guidelines. Additionally, we observed that ChatGPT lacks the ability to prioritize the relevance of clinical problems. In our study, when severe comorbidities, such as neurological impairments, were not explicitly emphasized in the prompt, the system tended to overlook them, focusing its attention on treating the tumor, which was the primary subject of the question. As a result, the model often recommended curative treatments, whereas the guidelines would indicate a palliative approach in such cases. In this context, it is essential to critically evaluate the information provided and assess its reliability; the user of generative AI is responsible for attributing meaning and determining the actionability of the generated content.

The issue of reliability of generative AI models will likely be mitigated by emerging techniques specifically designed to constrain the LLM answers to specific trusted knowledge. This is for example the case of Retrieval-Augmented Generation, a technique that is meant to augment the prompt provided to the LLM by enriching the query with relevant medical knowledge automatically retrieved from the parts of the knowledge (chunks) that are most pertinent with the original question.^25^ Additionally, most tools can now indicate the sources from which specific answer chunks are derived. Another promising development in the field of reliability and accuracy of AI models, is the introduction of multimodal LLMs (MLLMs), which are trained on different data modalities, such as text and images.^26^ Once trained on full DICOM-format datasets comprising millions of images and corresponding text reports, these models will likely demonstrate improved performance in biomedical image interpretation.

Another observation is that different LLMs can produce variable responses to the same prompt, highlighting the need for comparative analysis among them.^25,27^ Similarly, due to the way it is built, the model tends to align with the user’s tone, often responding in a manner that reflects the sentiment, if any, expressed in the prompt. A paradigmatic example was recently provided by Nobel Prize in Physics Giorgio Parisi during the National Academy of the Lincei conference *"Physical Roots of Modern AI"* on February 14, 2025. After initially receiving the correct answer from ChatGPT-4 for the calculation of 5 × 4, he repeatedly prompted the model in an attempt to mislead it, finally convincing it that 5 × 4 equals 25. This episode was reported in the media a few months ago. Given the continuous improvement of LLMs, it is likely that Professor Parisi would not be able to reproduce the same result today. Nevertheless, *AI hallucinations* still occur. Therefore, to maximize the chances of obtaining accurate and useful answers, medical users must learn how to interact effectively with the chatbot. AI systems rely solely on the data—such as texts and images—used during training, and unlike the human brain, they still lack complex interaction with the external world. This limitation reduces their current usefulness in enhancing multidisciplinary tumor board decisions, particularly in contexts where clinical guidelines are absent.^28^

Currently, no LLM-based tool has been recognized as a medical device, especially because of the lack of a standardized validation process, and this represents a significant unresolved issue given their growing use among physicians.^29^

Given all the considerations raised so far, it is very difficult to express a definitive opinion on the usefulness of these systems for diagnostic or therapeutic planning, as they are evolving so rapidly.

To conclude, we would like to reiterate that, in this editorial, we have critically discussed the use of generative AI in decision-making processes related to specific cancer patients. In contrast, there is no doubt that AI tools are highly effective in other areas of medical research and application. For example, drug discovery has greatly benefited from AI.^30^

AI will play an increasingly important role in scientific discovery in oncology. Assuming the computable nature of biological systems, the unique operational mechanisms of AI offer broad opportunities for generating knowledge from the interpretation of complex and seemingly disordered data—data that often defy traditional analytical tools. AI can integrate seemingly unrelated information, transforming what appears to be noise into a source of insight. What may seem to humans like random perturbation can be recognized and interpreted by AI systems, becoming a vehicle for meaning. Coming to the clinical context and clinical routine, tools now exist that can take notes and summarize discussions during meetings, translate clinical notes into different languages (which is particularly useful when traveling across countries), optimize appointment scheduling, and more. These applications, whose detailed description is beyond the scope of this editorial, do not directly influence decisions regarding a patient’s diagnosis or treatment, making them less critical from the ethical and regulatory standpoint.

As three leading physicists urged last year, scientists, governments, and citizens must come together in a large-scale international collaboration to safeguard humanity and ensure that the potential of AI serves everyone.^31^ Similarly, we expect the medical community to do the same, protecting itself from the misuse of AI through knowledge-sharing and by establishing international collaborations aimed at fully harnessing AI’s potential for cancer patients.

Ultimately, there is no domain of human knowledge that AI cannot explore, and there is nothing that humans can do that cannot, at least in principle, be made accessible to AI. The questions concerning the limits of AI usage are neither technological nor mathematical; they are ethical, and they should be addressed through appropriate regulatory and deontological measures.^32^ It therefore appears evident that a wise use of AI requires an ever-increasing collaboration, integration, and convergence of different and integrated disciplines.

## References

[1] European Union. Artificial Intelligence Act (AI Act): Regulation of the European Parliament and of the Council Laying Down Harmonised Rules on Artificial Intelligence. Regulation (EU) 2024/1156. Off J Eur Union 2024; 123: 1–95.

[2] Kulikowski CA. Beginnings of Artificial Intelligence in Medicine (AIM): Computational artifice assisting scientific inquiry and clinical art - with reflections on present AIM challenges. Yearb Med Inform 2019; 28: 249–256. 31022744 10.1055/s-0039-1677895 PMC6697545

[3] Shortliffe EH Davis R Axline SG , et al. Computer-based consultations in clinical therapeutics: explanation and rule acquisition capabilities of the MYCIN system. Comput Biomed Res 1975; 8: 303–320. 1157471 10.1016/0010-4809(75)90009-9

[4] Lanzola G Stefanelli M Barosi G. NEOANEMIA: a knowledge-based system emulating diagnostic reasoning. Comput Biomed Res 1990; 23: 560–582. 2276265 10.1016/0010-4809(90)90041-a

[5] Barosi G Magnani L Stefanelli M. Medical diagnostic reasoning: epistemological modeling as a strategy for design of computer-based consultation programs. Theor Med 1993; 14: 43–55. 8506539 10.1007/BF00993987

[6] Garber JR Patkar V. Computer-interpretable guidelines: electronic tools to enhance the utility of thyroid nodule clinical practice guidelines and risk stratification tools. Front Endocrinol (Lausanne) 2023; 14: 1228834. 37654563 10.3389/fendo.2023.1228834 PMC10465787

[7] Miles A Chronakis I Fox J , et al. Use of a computerised decision aid (DA) to inform the decision process on adjuvant chemotherapy in patients with stage II colorectal cancer: development and preliminary evaluation. BMJ Open 2017; 7: e012935. 10.1136/bmjopen-2016-012935 PMC5372112 28341685

[8] Meghiref Y Parnot C Duverger C , et al. The use of telemedicine in cancer clinical trials: Connect-patient-to-doctor prospective study. JMIR Cancer 2022; 8: e31255. 10.2196/31255 PMC8832259 34921544

[9] Lanzola G Polce F Parimbelli E , et al. The case manager: An agent controlling the activation of knowledge sources in a FHIR-based distributed reasoning environment. Appl Clin Inform 2023; 14: 725–734. 37339683 10.1055/a-2113-4443 PMC10499504

[10] Peleg M. Computer-interpretable clinical guidelines: a methodological review. J Biomed Inform 2013; 46: 744-763. 23806274 10.1016/j.jbi.2013.06.009

[11] Howe D Costanzo M Fey P , et al. Big data: The future of biocuration. Nature 2008; 455: 47–50. 18769432 10.1038/455047a PMC2819144

[12] Murphy SN Mendis M Hackett K , et al. Architecture of the open-source clinical research chart from Informatics for Integrating Biology and the Bedside. AMIA Annu Symp Proc 2007; 2007: 548-552. 18693896 PMC2655844

[13] Harris PA Taylor R Thielke R , et al. Research electronic data capture (REDCap)–a metadata-driven methodology and workflow process for providing translational research informatics support. J Biomed Inform 2009; 42: 377–381. 18929686 10.1016/j.jbi.2008.08.010 PMC2700030

[14] Simon GJ Aliferis C. Artificial Intelligence and Machine Learning in Health Care and Medical Sciences: Best Practices and Pitfalls. Cham: Springer, 2024. 39836790

[15] Pearl J . Theoretical impediments to machine learning with seven sparks from the causal revolution. In: Proc 11th ACM Int Conf Web Search Data Min. (WSDM ‘18) New York, NY: ACM; 2018, 3.

[16] Tran KA Kondrashova O Bradley A , et al. Deep learning in cancer diagnosis, prognosis and treatment selection. Genome Med 2021; 13: 152. 34579788 10.1186/s13073-021-00968-x PMC8477474

[17] Li M Jiang Y Zhang Y , et al. Medical image analysis using deep learning algorithms. Front Pub Health 2023; 11: 1273253. 38026291 10.3389/fpubh.2023.1273253 PMC10662291

[18] Clark P Kim J Aphinyanaphongs Y. Marketing and US Food and Drug Administration clearance of artificial intelligence and machine learning enabled software in and as medical devices: A systematic review. JAMA Netw Open 2023; 6: e2321792. 10.1001/jamanetworkopen.2023.21792 PMC10323702 37405771

[19] Parimbelli E Buonocore TM Nicora G , et al. Why did AI get this one wrong? - Tree-based explanations of machine learning model predictions. Artif Intell Med 2023; 135: 102471. 36628785 10.1016/j.artmed.2022.102471

[20] Salvi M Seoni S Campagner A , et al. Explainability and uncertainty: Two sides of the same coin for enhancing the interpretability of deep learning models in healthcare. Int J Med Inform 2025; 197: 105846. 39993336 10.1016/j.ijmedinf.2025.105846

[21] Devlin J Chang MW Lee K , et al. BERT: Pre-training of deep bidirectional transformers for language understanding. arXiv 2018; arXiv: 1810.04805.

[22] He K Mao R Lin Q , et al. A survey of large language models for healthcare: from data, technology, and applications to accountability and ethics. Inf Fusion 2025; 118: 102963.

[23] Cole D . The Chinese Room Argument. In: Zalta EN Nodelman U (eds), Metaphysics Research Lab, Stanford University, The Stanford Encyclopedia of Philosophy 2024.

[24] Premoli A Sacchi L Cadelli B , et al. Levering generative AI for decision support in early-stage breast cancer. New Technologies and Strategies to Fight Cancer. 9th Annual Meeting, Reggio Emilia, Italy, 28-30 November 2024.

[25] Fernández-Pichel M Pichel JC Losada DE. Evaluating search engines and large language models for answering health questions. NPJ Digit Med 2025; 8: 153. 40065094 10.1038/s41746-025-01546-w PMC11894092

[26] Li CY Chang KJ Yang CF , et al. Towards a holistic framework for multimodal LLM in 3D brain CT radiology report generation. Nat Commun 2025; 16: 2258. 40050277 10.1038/s41467-025-57426-0 PMC11885477

[27] Kahng M , et al. LLM Comparator: Interactive analysis of side-by-side evaluation of large language models. IEEE Trans Vis Comput Graph 2025; 31: 503-513. 39255096 10.1109/TVCG.2024.3456354

[28] Schmidl B Hütten T Pigorsch S , et al. Assessing the role of advanced artificial intelligence as a tool in multidisciplinary tumor board decision-making for recurrent/metastatic head and neck cancer cases – the first study on ChatGPT 4o and a comparison to ChatGPT 4.0. Front Oncol 2024; 14: 1455413. 39301542 10.3389/fonc.2024.1455413 PMC11410764

[29] Weissman GE Mankowitz T Kanter GP. Unregulated large language models produce medical device-like output. NPJ Digit Med 2025; 8: 148. 40055537 10.1038/s41746-025-01544-y PMC11889144

[30] Le MHN Nguyen PK Nguyen TPT , et al. An in-depth review of AI-powered advancements in cancer drug discovery. Biochim Biophys Acta Mol Basis Dis 2025; 1871: 167680. 39837431 10.1016/j.bbadis.2025.167680

[31] Baldi P Fariselli P Parisi G. Build an international AI 'telescope' to curb the power of big tech companies. Nature 2024; 634: 782. 10.1038/d41586-024-03436-9 39438746

[32] Bentzen SM. Artificial intelligence in health care: a rallying cry for critical clinical research and ethical thinking. Clin Oncol (R Coll Radiol) 2025; 41: 103798. 40184826 10.1016/j.clon.2025.103798

